# Dynamics of autonomic nervous system responses and facial expressions to odors

**DOI:** 10.3389/fpsyg.2014.00110

**Published:** 2014-02-13

**Authors:** Wei He, Sanne Boesveldt, Cees de Graaf, René A. de Wijk

**Affiliations:** ^1^Consumer Science and Intelligent Systems, Food and Biobased Research, Wageningen University and Research CentreWageningen, Netherlands; ^2^Division of Human Nutrition, Sensory Science and Eating Behaviour, Wageningen UniversityWageningen, Netherlands

**Keywords:** skin conductance, skin temperature, heart rate, ANS responses, odor, valence, concentration, facial expressions

## Abstract

Why we like or dislike certain products may be better captured by physiological and behavioral measures of the autonomic nervous system (ANS) than by conscious or classical sensory tests. Responses to pleasant and unpleasant food odors presented in varying concentrations were assessed continuously using facial expressions and responses of the ANS. Results of 26 young and healthy female participants showed that the unpleasant fish odor triggered higher heart rates and skin conductance responses, lower skin temperature, fewer neutral facial expressions and more disgusted and angry expressions (*p* < 0.05). Neutral facial expressions differentiated between odors within 100 ms, after the start of the odor presentation followed by expressions of disgust (180 ms), anger (500 ms), surprised (580 ms), sadness (820 ms), scared (1020 ms), and happy (1780 ms) (all *p*-values < 0.05). Heart rate differentiated between odors after 400 ms, whereas skin conductance responses differentiated between odors after 3920 ms. At shorter intervals (between 520 and 1000 ms and between 2690 and 3880 ms) skin temperature for fish was higher than that for orange, but became considerable lower after 5440 ms. This temporal unfolding of emotions in reactions to odors, as seen in facial expressions and physiological measurements supports sequential appraisal theories.

## Introduction

Up to 80% of all new food products fail in the marketplace, despite the fact that they are typically subjected to a large number of sensory and consumer tests before their market introduction (Crawford, 1977). This suggests that the “standard” sensory and consumer tests, which typically include sensory analytical profiling and liking tests, have a low predictive validity with respect to general product performance. Possibly, consumer food choice outside the laboratory may be less based on cognitive information processing and rational reasoning, and more on unarticulated/unconscious motives and associations (Wansink, 2004). Reasons for likes or dislikes of specific foods are typically difficult to articulate but may determine much of our food choice. Unarticulated/unconscious motives and associations are not very well captured by traditional tests based on conscious cognitive processes, and may be better captured by physiological and behavioral measures (e.g., facial expressions) of the autonomic nervous system (ANS) which do not require conscious processes (Greenwald, 2009).

Physiological measures have been used extensively to capture responses of the ANS to various types of stimuli such as film clips, personalized recall of specific situations, and odors. In a previous study, Alaoui-Ismaïli et al. (1997) related various autonomic parameters to the pleasantness of five odorants, and found that unpleasant odors were associated with increased heart rate (HR) and longer skin conductance responses (SCR) compared to pleasant odors. Bensafi et al. (2002) related ANS measures to rated pleasantness, arousal, intensity, and familiarity for a set of six odorants and found that their results could be explained by two main factors: pleasantness, inversely related to HR (similar to Alaoui-Ismaïli et al., 1997) and arousal, positively related to skin conductance and rated intensity. Delplanque et al. (2009) found stronger SCR and higher HR for unpleasant compared to pleasant odors. They also established that HR differences between pleasant and unpleasant odors occurred relatively late in the deceleration phase, approximately 5–8 s after odor presentation. Considerable faster odor-specific responses were found for facial expressions; facial muscle activity associated with positive and negative facial expressions showed different activities for pleasant and unpleasant odors as soon as 400–500 ms after odor presentation (Delplanque et al., 2009).

Facial expressions have also been used extensively by others to measure emotional responses to food-related stimuli. Well-known are the positive facial expressions of new-borns toward liked (sweet) and the negative expressions toward disliked (bitter) basic tastes, extensively documented by Steiner (1973). More recently, an automated tool, FaceReader, has been developed and used to analyze more diverse, universal facial expressions. Using different food stimuli, it was found that happy expressions were not systematically related to liking scores, in contrast to neutral, angry, and disgusted expressions (Danner et al., 2014), and that stronger facial expressions to disliked foods compared to liked foods were already detected at the first visual encounter with the food (De Wijk et al., 2012).

The dynamic features over time of physiological responses and facial expressions have typical been outside the scope of most studies, even though they play a key role in several modern theories on emotion, the so-called componential appraisal models (see Ellsworth and Scherer, 2003 for an overview). The models assume that the elicitation and the differentiation of emotions are determined by appraisals, the continuous, recursive evaluations of events, Delplanque et al. (2009) investigated the appraisal of odor novelty and pleasantness and consequent emotional responses by measuring facial muscle activity and HR. They demonstrated that odors were detected as novel or familiar before being evaluated as pleasant or unpleasant (Distel et al., 1991; Royet et al., 1999). In addition, their results also argued in favor of a dynamic construction of facial expressions providing support for sequential appraisal theories (Ellsworth and Scherer, 2003). For example, early reactions, such as raising the eyebrows and opening the eyes, were related to the detection of a novel or unexpected stimulus, which is associated with increased alertness and attention. After this novelty detection, assessment of pleasantness may lead to avoidance when the stimulus is aversive or threatening, or approach when a pleasant response is activated.

The present study will expand on previous studies by using (food) odors delivered by an olfactometer, offering a high degree of control over timing and concentrations, and by incorporating additional ANS measures [skin temperature (ST)] and other types of facial expressions. Similar to Delplanque et al. (2009) the present study will also focus on the temporal development of each measure instead of the more commonly used time-averaged means (e.g., De Wijk et al., 2012; Danner et al., 2014). Physiological responses and facial expressions will be measured continuously and analyses will be based on time-averaged means (similar to most of the previous studies) as well as on their temporal development. It is hypothesized that the results based on time-averaged means will replicate the findings of similar studies by others, i.e., higher HR and skin conductance, lower ST and more negative facial expressions after exposure to the unpleasant odor compared to exposure to the pleasant odor. It is further hypothesized that ANS responses are slower than facial expressions, but that both follow sequential appraisal processes of evaluating the stimuli.

## Materials and methods

### Participants

Twenty-six young healthy female participants (mean age: 22.6 ± 1.5 years, range: 20–25 years, 18.5 < BMI < 25 kg/m^2^) were recruited from the subject pool of Food and Biobased Research, part of Wageningen University and Research Center. Participants self-reported their BMI and if they had actual/previous history of smell or taste disorders known to affect chemosensory function. Detailed information regarding the experiment was given and an informed consent form was signed by all participants prior to testing. The study was approved by the Medical Ethical Committee of the Wageningen University.

### Odor stimuli and presentation

As described elsewhere (He et al., under review), two food odors were selected on the basis of their relatively negative (fish odor) or positive (orange odor) valence (Boesveldt et al., 2010). The orange (cold-pressed Californian orange oil, Sigma Aldrich, St. Louis, MO, USA) and fish (Fish flavor oil, Givaudan Inc., Geneva, Switzerland) odors were diluted with mineral oil (to 70%, v/v) and 1,2-propanediol (to 27%, v/v), respectively. With a dynamic olfactometer based on air-dilution (OM2s, Burghart instruments, Wedel, Germany), each odor was delivered in three different concentrations (low, medium, or high), correspondingly perceived at different intensities in a pilot study. The olfactometer allows the presentation of odorous stimuli within a continuous humidified (80%) and warmed (37°C) airstream of 8 L/min, which does not alter the mechanical or thermal conditions at the nasal mucosa (Kobal and Hummel, 1988). These stimuli were delivered through a nosepiece for 1 s with an inter stimulus interval of 60 s. Each block of six stimuli (i.e., orange odor in three concentrations and fish odor in three concentrations) was randomized and presented five times, for a total of 30 stimuli.

### Procedure

**Figure 1:**
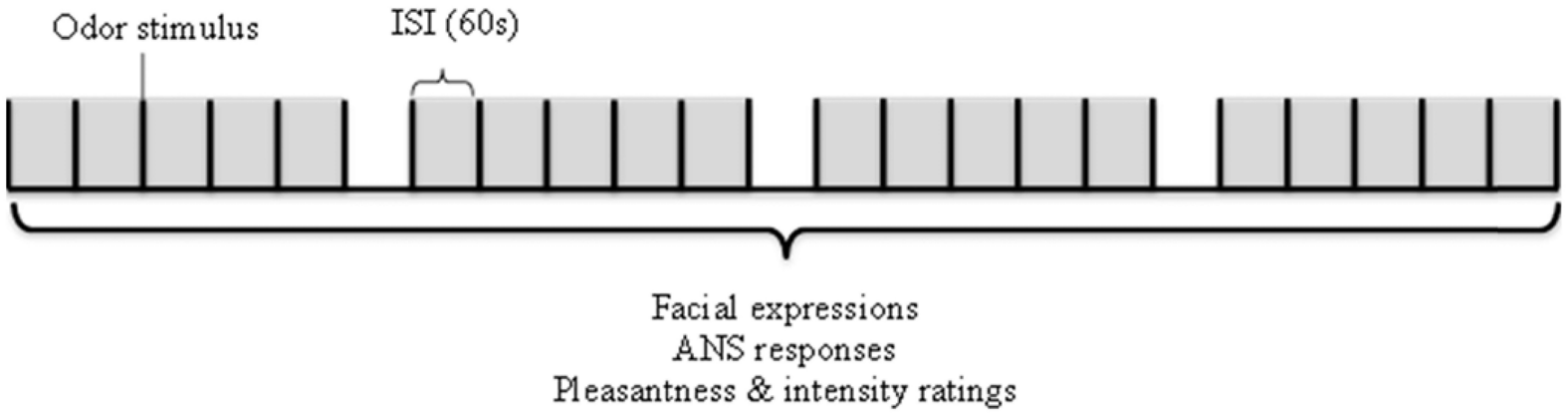
**Schematical representation of the experimental procedure followed during one experimental session**.

The experimental sessions took place in the physiological laboratory of the Restaurant of the Future located in Wageningen, the Netherlands. The experiment leader explained the experiment to the participant, allowed ample time for questions and asked the participant to sign the inform consent form (which they had received by e-mail prior to the experimental session) after which the electrodes were placed. Participants were seated in a comfortable chair, fitted with the olfactometer nosepiece, and oriented toward an adjustable computer monitor set with a webcam at eye-level (1 m viewing distance). They were asked to look directly toward the camera while receiving the odor stimulus to ensure recognition by the FaceReader software. Each trial started with an auditory attention signal to remind the participant to pay attention to the upcoming odor. The pleasantness and intensity of each odor was rated subsequently on a paper questionnaire 10 s after stimulation. The procedure is also shown schematically in Figure 1. The whole experiment lasted 45 min in total. Photograph 1 shows the set-up as used in this study.

**Photograph 1:**
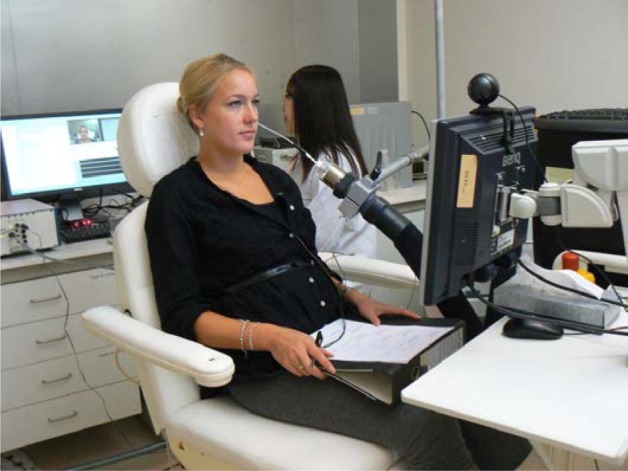
**Set-up used in this study showing the participant and the experimenter, the arm of the olfactometer for odor presentation, and the monitor used for instructions with a camera used for facial expressions**.

### Measurements

#### Physiological ANS measures

Physiological measures included:

1. Skin conductance response (SCR) measured in μSiemens with electrodes placed on the palm of the non-dominant hand of the participant.
2. Heart rate (HR) measured in beats per minute with electrodes placed on the chest.
3. Skin temperature (ST) measured in degrees Celsius with an electrode placed on the palm of the non-dominant hand of the participant.

The physiological data were collected at 200 Hz via a MindWare Acquisition data acquisition system (MindWare Technologies, Inc.) with separate filter settings for the electrocardiogram, finger temperature and electrodermal (SCR) activity. Filter settings were low-pass 0.5 Hz, high-pass 45 Hz for HR frequency, low-pass 1 Hz, high-pass 45 Hz for SCR, and low-pass 10 Hz, high-pass 45 Hz for ST. Electrodes were used with a surface of 4.1 cm^2^ and filled with 1% Chloride wet gel. Signals were transferred to the Acquisition Unit (16-bit A/D conversion) and stored on computer hard disk (sampling rate 500 Hz/s). Electrocardiographic R waves were detected offline, and intervals between heartbeats were converted to HR, expressed in beats per minute (BPM). SCR activity was recorded (high-pass filter: 0.025 Hz.) by the constant voltage method (0.5 V). The signal was amplified by 1000 and low-pass filtered (30 Hz).

#### Facial expressions

Facial expressions were automatically analyzed using FaceReader software version 4.0 (Noldus Information Technology B.V.). FaceReader works in three steps: (1) face finding, (2) face modeling, and (3) face classification. During face finding an accurate position of the face is found using the Active Template Method. During modeling, the Active Appearance Model is used to synthesize an artificial face model, which describes the location of 491 key points as well as the texture of the face. The actual classification of the facial expressions is done by training an artificial neural network as training material nearly 2000 manually annotated images were used. The network was trained to classify the six basic or universal emotions described by Ekman (1992): happy, sad, angry, surprised, scared, and disgusted and a neutral state. FaceReader analyzed the facial expressions on a frame-by-frame basis, i.e., at 25 Hz. Previous studies showed that FaceReader results corresponded between 71% (angry) to 99% (neutral) of all cases, with an average of 87%, with results from human observers (Terzis et al., 2012). FaceReader happiness scores correlated significantly (*r* = 0.79) with objectively measured activity in the *zygomaticus supercilli* or cheek muscle, a muscle that is activated during expressions of happiness (D'Arcey et al., 2012). A more detailed description of the science behind FaceReader can be found at: http://info.noldus.com/free-white-paper-on-facereader-methodology/.

#### Ratings of pleasantness and intensity

A visual analog scale of 10 cm was used to rate pleasantness and intensity after each odor presentation, ranging from “not perceivable” (left-hand end = 0 cm) to “extremely strong” (right-hand end = 10 cm), or from “very unpleasant” (left) to “neutral” (middle of the scale = 5 cm) to “very pleasant” (right). In this study, orange odors were rated more pleasant [*F*_(1, 25)_ = 99.86, *p* < 0.001] and less intense [*F*_(1, 25)_ = 17.27, *p* < 0.001] than fish odors by the participants (see Table 1). Furthermore, odor intensity increased with concentration [*F*_(2, 50)_ = 47.15, *p* < 0.001].

**Table 1:** **Average ratings (0–10, with standard deviation) of fish and orange odors diluted to different concentrations**.

**Odor**	**Concentration**	**Air-diluted to (%)**	**Intensity**		**Pleasantness**	
			**Mean**	***SD***	**Mean**	***SD***
Fish (27% v/v)	Low	10	6.2	1.9	1.5	1.3
	Medium	25	6.7	1.6	1.5	1.3
	High	50	7.1	1.7	1.3	1.1
Orange (70% v/v)	Low	50	4.8	1.7	6.4	1.1
	Medium	80	6.0	1.7	5.6	1.6
	High	100	6.6	1.7	5.4	1.3

Ratings were made on a visual analog scale of 10 cm length. For intensity, 0 indicates “not perceivable” and 10 indicates “extremely strong”; For pleasantness, 0 indicates “very unpleasant,” 5 indicates “neutral,” and 10 indicates “very pleasant.”

### Data analysis

The processed images with the facial expressions were combined with raw physiological data in Observer XT 10.5 software (Noldus Information Technology) for further analyses. The moments that odors were presented to the participants were marked automatically using the “trigger-out” signal from the olfactometer that signals the start of each odor presentation. The physiological measures SCR, HR, and ST were analyzed per odor presentation. The video images of the facial expressions were processed per odor presentation with FaceReader 4.0 software (Noldus Information Technology). Due to a technical malfunction, absolute ST values were not recorded, but the results can still be used to assess changes over time in ST per odor presentation. Results from some participants had to be removed from the analysis due to a large number of artifacts. The number of participants that is included in the analysis is 21 (HR), 22 (skin conductance and ST), and 24 (facial expressions).

Two types of statistical analyses were used: one based on post-odor time-averaged responses to verify systematic effects of odor and concentration, and one based on pre- and post-odor time-series of responses to verify the post-odor time at which responses become odor-specific. Details of each type of analysis are given below. In addition, correlational analysis was used to verify systematic associations between measures.

1. Repeated measures ANOVAs (IBM SPSS Statistics 19.0, IBM Corporation, Armonk, USA) were conducted on post-odor time-averaged facial expressions, ANS responses with odor and concentration as within-subject variables. A *p*-value of 0.05 was considered significant.
2. To verify the time at which time-series ANS responses and facial expressions become odor-specific (i.e., differ significantly between odors), absolute deltas between orange and fish odors were calculated together with the standard deviations for the 2.5 s interval preceding odor presentation to establish a pre-odor baseline. Subsequently, post-odor times were identified at which the absolute delta between the odors exceeded the pre-odor average plus three times the pre-odor standard deviation.

## Results

### Effects of odor and concentration

#### Physiological measures

**Figure 2:**
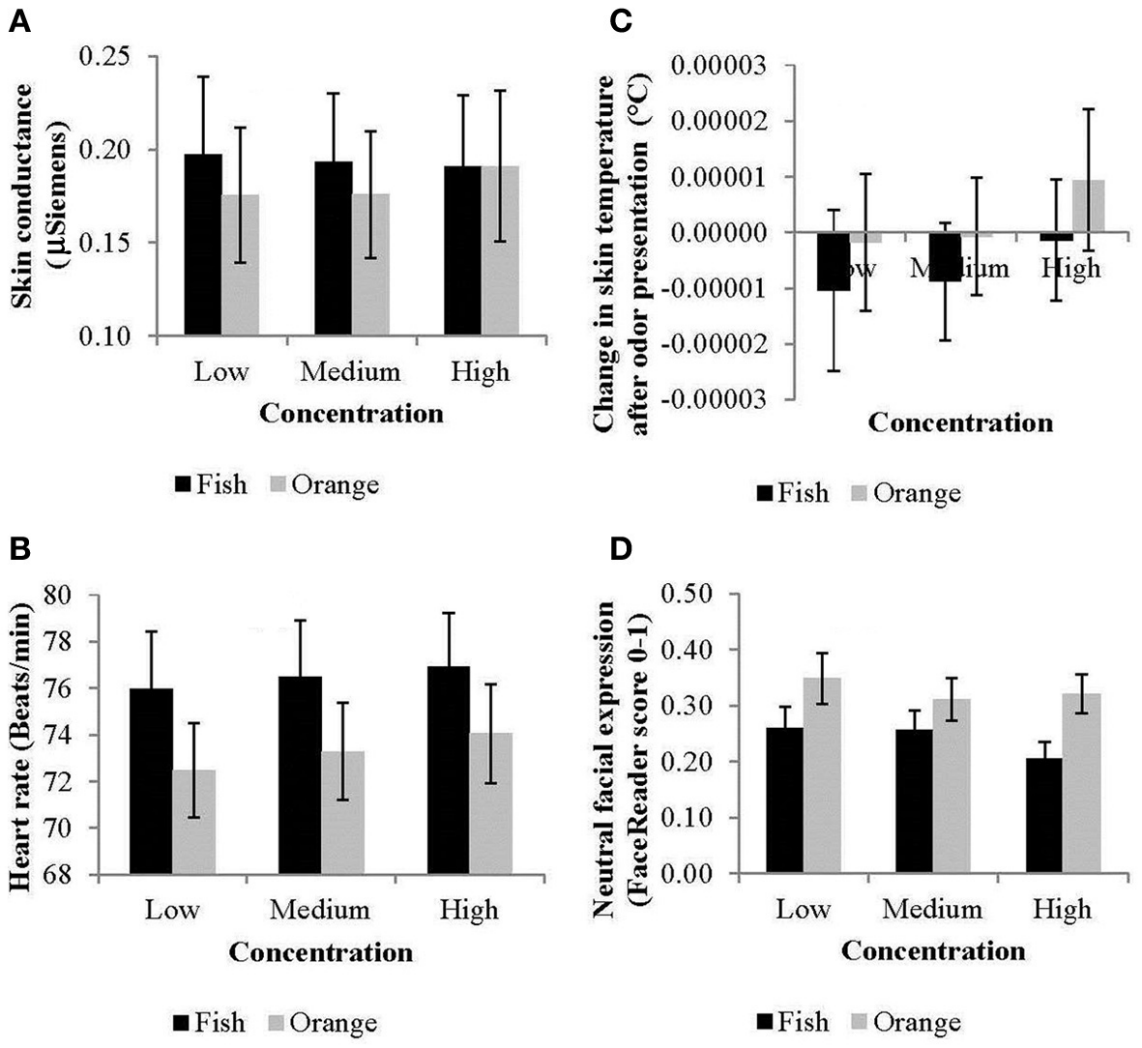
**Effects of odor and concentration on (A) skin conductance responses, (B) heart rate, (C) skin temperature, and (D) neutral facial expressions (averaged across time and bars indicate standard errors)**.

Time-averaged means for HR [*F*_(1, 20)_ = 18.7, *p* < 0.001] and skin conductance [*F*_(1, 21)_ = 6.3, *p* < 0.05] were significantly higher for the unpleasant fish odor compared to the pleasant orange odor (Figures 2A,B). Skin temperature did not vary systematically with odor [*F*_(1, 21)_ = 2.0, n.s.; Figure 2C]. Heart rate also increased systematically with concentration [*F*_(2, 40)_ = 5.3, *p* < 0.01]. Concentration did not affect skin conductance [*F*_(1, 21)_ = 0.6, n.s.] or ST *F*_(1, 21)_ = 0.9, n.s.).

#### Facial expressions

Time-averaged means of facial expressions to the fish compared to the orange odor were significantly less neutral [*F*_(1, 23)_ = 21.25, *p* < 0.001; Figure 2D] and more disgusted [*F*_(1, 23)_ = 9.63, *p* < 0.01], and angry [*F*_(1, 23)_ = 4.00, *p* < 0.05]. Moreover, facial expressions intensified at higher concentrations resulting, depending on the odor, in weaker neutral expressions [odor by concentration effect: *F*_(2, 46)_ = 3.25, *p* < 0.05] and stronger scared expressions [odor by concentration effect: *F*_(2, 46)_ = 3.51, *p* < 0.05].

Associations between physiological measures, facial expressions, and ratings are summarized by correlational analysis based on 24 stimuli (two odors × three concentrations × four replicates) averaged across participants (Table 2).

**Table 2:** **Pearson correlation coefficients between facial expressions, ratings and physiological measures for 24 stimuli averaged across participants**.

		**Facial expressions**							**Ratings**		**Physiological measures**		
		Angry	Disgusted	Happy	Neutral	Sad	Scared	Surprised	Pleasantness	Intensity	HR	SCR	ST
Facial expressions	Angry	1	,49	,61^*^	−,65^*^	0.07	,50	−0.36	−,71^*^	,49	,61^*^	−0.09	0.02
	Disgusted		1	,40	−,78^*^	,48	,57^*^	−,59^*^	−,71^*^	,56^*^	,61^*^	0.04	−0.02
	Happy			1	−,52^*^	−0.04	0.31	−0.18	−,55^*^	,57^*^	,50	−0.21	0.12
	Neutral				1	−,43	−,58^*^	,48	,83^*^	−,55^*^	−,73^*^	0.15	0.19
	Sad					1	,43	−0.33	−,41	,41	,45	,56^*^	−0.23
	Scared						1	−0.23	−,61^*^	,52^*^	,50	0.06	−0.10
	Surprised							1	,65^*^	−0.35	−,62^*^	−0.22	−0.03
Ratings	Pleasantness								1	−,61^*^	−,93^*^	−0.08	0.21
	Intensity									1	,67^*^	0.07	0.17
Physiological measures	HR										1	0.23	−0.16
	SCR											1	0.11
	ST												1

* Correlation is significant at the 0.005 level (2-tailed).

### Time-series responses: when do responses become odor specific?

#### Physiological measures

**Figure 3:**
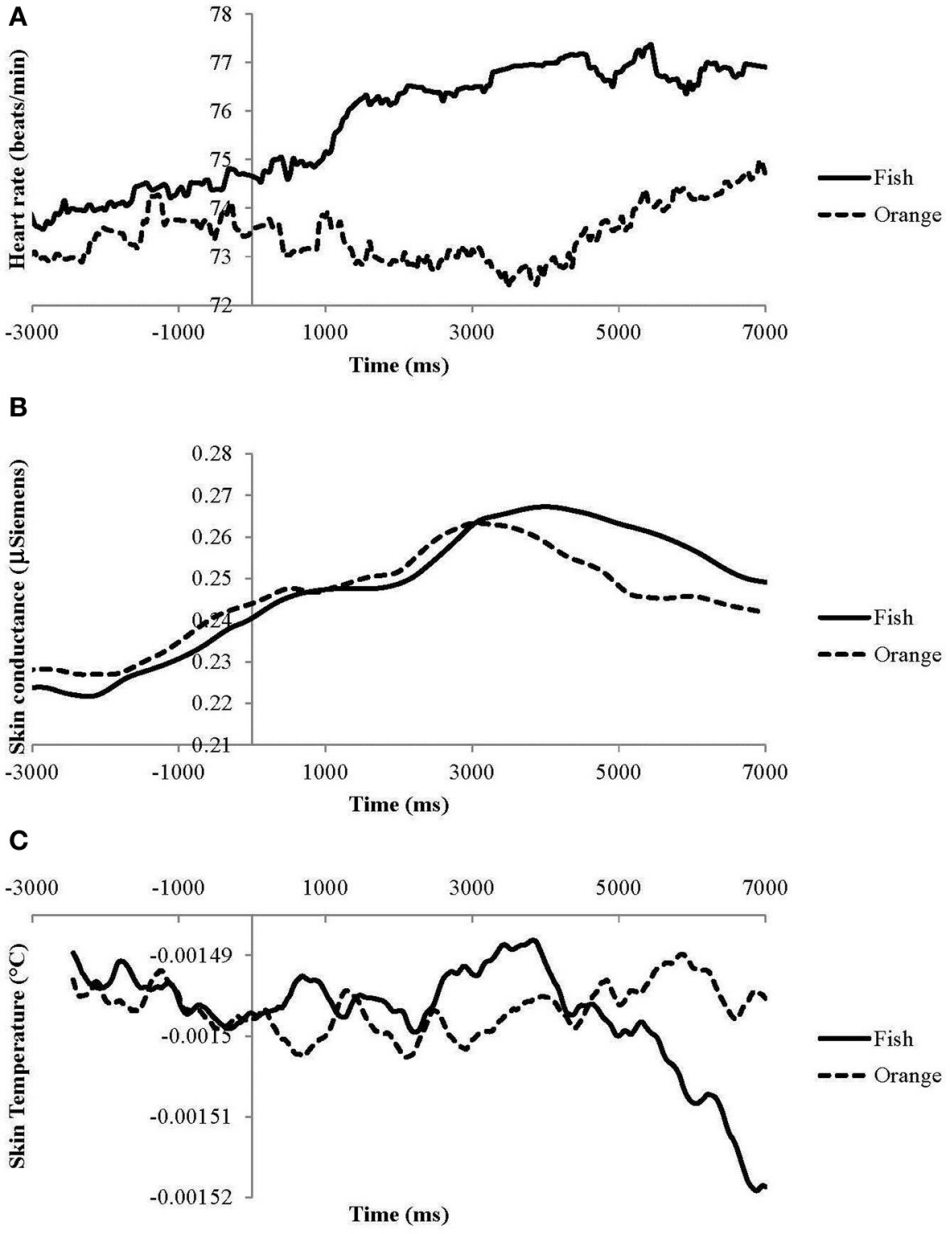
**Effects of odor (averaged across concentrations) on (A) heart rate, (B) skin conductance, and (C) skin temperature. Absolute skin temperatures are incorrect due to a technical malfunction**.

Prior to the odor presentation, but after the warning signal is given, ANS measures show gradual changes that are independent of the odor valence whereby skin conductance and HR gradually increase and ST gradually decreases (Figure 3). Skin conductance continues to increase for seconds after odor presentation independent of the specific odor. After approximately 3 s, SCR for orange decreases whereas that for fish continues to increase. The difference in SCR becomes significant after 3920 ms (Figure 3B and Table 3). Heart rate for the unpleasant fish odor increases almost instantaneously after the odor is presented whereas HR for the pleasant orange odor shows much smaller effects (Figure 3A and Table 3). The difference in HR response between the odors becomes significant after 400 ms. Skin temperature follows a different, irregular pattern with higher temperatures for fish odor at shorter intervals (between 520 and 1000 ms and between 2690 and 3880 ms) and lower temperature at longer intervals (after 5440 ms) (Figure 3C and Table 3) compared to orange odor.

**Table 3:** **Intervals in ms following odor presentation at which responses become odor-specific**.

**Measures**		**Odor-specific time (ms)**
Facial expressions	Neutral	<100-end
	Happy	1780-end
	Sad	820-end
	Angry	500-end
	Surprised	580-end
	Scared	1020-end
	Disgusted	180-end
ANS responses	HR	400-end
	SCR	3920-end
	ST	520–1000
		2640–3880
		5440-end

#### Facial expressions

**Figure 4:**
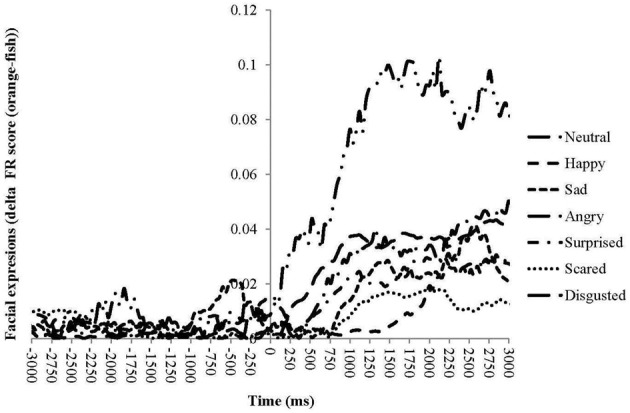
**Sequential unfolding of differences in facial expressions between the unpleasant fish odor and the pleasant orange odors for seven emotional facial expressions over time following the odor presentation**.

Neutral expressions become odor-specific after less than 100 ms. Disgusted expressions take approximately another 100 ms to become odor-specific. Angry, surprised, sad, and scared become after 500–1000 ms odor-specific, whereas happy expression become odor-specific after more than 1700 ms (Figure 4 and Table 3). Table 3 summarizes the times at which ANS responses and facial expressions significantly differentiate between the unpleasant fish and pleasant orange odor.

## Discussion

Human responses to pleasant and unpleasant food odors presented in varying concentrations were assessed with facial expressions and responses of the ANS. Analysis were carried out on results with and without averaging over time, and showed partly overlapping and partly different results.

ANOVAs on time-averaged results showed that the unpleasant fish odor triggered higher HR and SCR, lower ST, fewer neutral facial expressions and more disgusted and angry expressions compared to the pleasant orange odor. Overall, our results were similar to the ones found in studies by others for HR (Alaoui-Ismaïli et al., 1997; Bensafi et al., 2002; Delplanque et al., 2009), skin conductance (Alaoui-Ismaïli et al., 1997; Delplanque et al., 2009), and ST (see Köster, 2009), indicating that these averaged physiological measurements are mainly responsive to the valence of a stimulus, and less to intensity, whereas facial expressions appear to demonstrate more concentration-specific effects.

Correlational analyses based on time-averaged results shows positive associations between odor liking and neutral/surprised facial expressions, and negative associations between odor liking and all other facial expressions, including happiness. Negative associations between odor liking and happy facial expressions have also been reported previously by others (Zeinstra et al., 2009; Danner et al., 2014; He et al., under review) suggesting that happy expressions cannot discriminate liked or disliked foods implicitly. Facial expressions of happiness are rarely displayed when one is alone and social interactions are absent suggesting that these expressions serve a social function (see also Gilbert et al., 1987 and Parkinson, 2005). The fact that they did occur in this study in the presence of experimental staff suggests that the happy facial expressions may serve some kind of social signaling function, e.g., to signal the staff that one is OK despite the previous display of negative expressions associated with disliked odors.

When results are not averaged across time, analyses demonstrate that facial expressions and physiological responses become rapidly odor-specific and are dynamic in nature. Responses such as skin conductance already start before the actual odor presentation These responses are obviously odor non-specific and probably reflect anticipatory processes, Almost immediately after the onset of the odor presentation, neutral facial expressions decrease followed after 100 ms by an increase in facial expressions of disgust. Within 400 ms HR for the unpleasant odor increase (similar to rapid acceleration in HR observed for negative emotions by Levenson, 1988), ST briefly increases, followed between 500 and 1000 ms by facial expressions of angry, surprised, sad, and scared, and after 1700 ms by happy expressions. During all this time, skin conductance gradually increases for both odors until approximately 3 s when skin conductance for the pleasant odor starts to decrease whereas that for the unpleasant odor continues to increase. Finally, after more than 4 s, skin conductance for the unpleasant odor decreases together with ST for the unpleasant odor. Combined these time-related results show that most facial expressions and physiological responses are fast reacting and odor-specific.

Our results correspond well with those found in previous studies; Delplanque et al. (2009) found odor-specific activities in two types of facial muscle activities 400–500 ms after odor presentation, which coincides approximately with sad, angry, and surprised expressions in the present study. These values also concur with the values found for other stimulus modalities such as vision; Dimberg et al. (2002) found facial responses to positive or negative visual stimuli after approximately 400–500 ms. In addition, we found other expressions that were triggered even faster, such as disgust, or slower, such as happy.

Response times for HR and for most of the facial expressions are well within 1 s after the odor is presented, and are often shorter than for example response time for odor detection (approximately 800 ms, De Wijk, 1989) or response time to decide whether or not an odor is more pleasant than a previous one (approximately 850 ms, Olofsson et al., 2012), where conscious action is needed. These differences in timing are possibly related to automated vs. conscious processes in the central nervous system. Facial expressions and ANS responses probably reflect automated processing of the central nervous system (see Dimberg et al., 2002 for automated processes and facial expressions), whereas decisions regarding detection and pleasantness/unpleasantness require also time-consuming conscious processing. The fact that automated emotional odor-response times may be as fast as response times in the visual domain despite the relatively slow peripheral and peri-peripheral processing of odors may reflect the anatomical overlap between CNS structures involved in olfaction and emotions; the peripheral and central olfactory system are only separated by one relay (glomerulus of the olfactory bulb) after the odor interacts with the primary olfactory neurons. Next, olfactory information is conducted to other olfactory structures, some of which are also involved in emotions (hippocampus, anterior cingulate cortex, orbitofrontal cortex and parts of the amygdala and insula Lundström et al., 2011; Soudry et al., 2011). Given the close correspondence of CNS structures involved in olfaction and emotions and the fact that these structures are activated simultaneously to when information becomes available for conscious, higher order cognitive processing in the cortex, it is no longer surprising that automated emotional odor response times are often faster than odor response times that involve conscious processing.

Combined the time-series responses found in this study show that most facial expressions and physiological responses are fast reacting and odor-specific. Moreover, different facial expressions and physiological measures develop at their own specific rate over time. Consequently, responses to the same stimulus may produce very different patterns of results depending on the time at which they are assessed. For example, fast responses around 500 ms, may be dominated by negative facial expressions such as disgust, increased HR and increased ST, whereas slower responses may be dominated by positive facial expressions, lower HR and decreased ST. The fast responses may be automated reflexes to novel and potentially dangerous stimuli, as observed by Delplanque et al. (2009), whereas the later responses may reflect a conscious processing of a sequence of different emotions, each resulting from a different appraisal of the stimulus by the observer (e.g., Ellsworth and Scherer, 2003). Results from the same laboratory indicate that conscious evaluative ratings of participants are associated with ANS responses and facial expressions between one and three seconds after stimulation (He et al., under review). This supports the notion that the fast responses, with response times of less than one second, are automated and relatively independent of evaluative ratings, whereas slower responses reflect conscious processing that form the basis for evaluative ratings and facial expressions of happiness for communicative purposes.

The present study has its obvious limitations; only a small number of odors were investigated, and their effects were investigated under controlled laboratory conditions with female participants. Nevertheless, the results may have some implications for consumer behavior in the real world. For example, visitors to supermarkets may have approximately 45 min to select their weekly groceries from up to 30,000 products. This task becomes even more daunting considering the fact that many of these selections are not planned but made in the supermarket. Given this abundance of choices consumers need a fast and partly automated selection mechanism that combines affect, appraisal, action readiness and autonomic arousal. This fast selection mechanism may be based on fast and probably automated ANS responses and facial expressions similar to the ones found in the present study. These fast responses may not only be triggered by odors, but also product packages and brand names. To explore real-life applications, future studies will measure ANS responses and facial expressions in relation to consumer choice behavior. Initially, consumer behavior will be assessed in the semi-real-life test environment of a virtual supermarket, followed by real-life assessment in an actual supermarket. Such studies will allow a proper evaluation of ANS measures and facial expressions as tools for marketing (research) because their associations with consumer product interactions and purchasing behaviors will be tested directly.

In summary, physiological and facial responses to odors prove to be fast and dynamic and the balance between these responses is continuously changing depending on their timing. This changing balance may reflect different sequential appraisals of emotions. This study along with other recent studies (e.g., Delplanque et al., 2009) shows the necessity of taking the time dimension into account and future studies should further explore the relation between dynamic responses and appraisals.

### Conflict of interest statement

The authors declare that the research was conducted in the absence of any commercial or financial relationships that could be construed as a potential conflict of interest.

## References

[B1] Alaoui-Ismaïli O. Vernet-Maury E. Dittmar A. Delhomme G. Chanel J. (1997). Odor hedonics: connection with emotional response estimated by autonomic parameters. Chem. Senses 22, 237–248 10.1093/chemse/22.3.237 9218136

[B2] Bensafi M. Rouby C. Farget V. Bertrand B. Vigouroux M. Holley A. (2002). Autonomic nervous system responses to odours: the role of pleasantness and arousal. Chem. Senses 27, 703–709 10.1093/chemse/27.8.703 12379594

[B3] Boesveldt S. Frasnelli J. Gordon A. R. Lundström J. N. (2010). The fish is bad: negative food odors elicit faster and more accurate reactions than other odors. Biol. Psychol. 84, 313–317 10.1016/j.biopsycho.2010.03.006 20227457

[B4] Crawford C. (1977). Marketing research and the new product failure rate. J. Marketing 41, 55–61 10.2307/1250634

[B5] Danner L. Sidorkina L. Joechl M. Duerrschmid K. (2014). Make a face! Implicit and explicit measurement of facial expressions elicited by orange juices using face reading technology. Food Qual. Prefer. 32, 167–172 10.1016/j.foodqual.2013.01.004

[B6] D'Arcey T. Johnson M. Ennis M. (2012). Assessing the validity of FaceReader using facial electromyography, in Proceedings of APS 24th annual meeting (Chicago, IL).

[B7] Delplanque S. Grandjean D. Chrea C. Coppin G. Aymard L. Cayeux I. (2009). Sequential unfolding of novelty and pleasantness appraisals of odors: evidence from facial electromyography and autonomic reactions. Emotion 9, 316–328 10.1037/a0015369 19485609

[B8] De Wijk R. A. (1989). Temporal Factors in Human Olfactory Perception. Doctoral dissertation, State University of Utrecht, The Netherlands

[B9] De Wijk R. A. Kooijman V. Verhoeven R. H. G. Holthuysen N. T. E. de Graaf C. (2012). Autonomic nervous system responses on and facial expressions to the sight, smell, and taste of liked and disliked foods. Food Qual. Prefer. 26, 196–203 10.1016/j.foodqual.2012.04.015

[B10] Dimberg U. Thunberg M. Grunedal S. (2002). Facial reactions to emotional stimuli: automatically controlled emotional responses. Cogn. Emot. 16, 449–471 10.1080/0269993014300035615652310

[B11] Distel H. Ayabe-Kanamura S. Martínez-Gómez M. Schicker I. Kobayakawa T. Saito S. (1991). Perception of everyday odors—correlation between intensity, familiarity and strength of hedonic judgement. Chem. Senses 24, 191–199 10.1093/chemse/24.2.191 10321820

[B12] Ekman P. (1992). Facial expressions of emotion: an old controversy and new findings. Philos. Trans. R. Soc. Lond. B Biol. Sci. 335, 63–69 10.1098/rstb.1992.0008 1348139

[B13] Ellsworth P. C. Scherer K. R. (2003). Appraisal processes in emotion, in Handbook of Affective Sciences, eds Davidson R. J. Scherer K. R. Goldsmith H. H. (New York, NY: Oxford University Press), 572–595

[B14] Gilbert A. N. Fridlund A. J. Sabini J. (1987). Hedonic and social determinants of facial displays to odors. Chem. Senses 12, 355–363 10.1093/chemse/12.2.3558055269

[B15] Greenwald A. (2009). Supplemental material for understanding and using the implicit association test: III. Meta-analysis of predictive validity. J. Pers. Soc. Psychol. 97, 17–41 10.1037/a0015575 19586237

[B17] Kobal G. Hummel C. (1988). Cerebral chemosensory evoked potentials elicited by chemical stimulation of the human olfactory and respiratory nasal mucosa. Electroencephalogr. Clin. Neurophysiol. 71, 241–250 10.1016/0168-5597(88)90023-8 2454788

[B18] Köster E. (2009). Diversity in the determinants of food choice: a psychological perspective. Food Qual. Prefer. 20, 70–82 10.1016/j.foodqual.2007.11.002

[B19] Levenson R. (1988). Emotion and the autonomic nervous system: a prospectus for research on autonomic specificity, in Social Psychophysiology and Emotion: Theory and Clinical Applications, ed Wagner H. L. (London: John Wiley and Sons), 17–42

[B19a] Lundström J. N. Boesveldt S. Albrecht J. (2011). Central processing of the chemical senses: an overview. ACS Chem. Neurosci. 2, 5–16 10.1021/cn1000843 21503268 PMC3077578

[B20] Olofsson J. K. Bowman N. E. Khatibi K. Gottfried J. A. (2012). A time-based account of the perception of odor objects and valences. Psychol. Sci. 23, 1224–1232 10.1177/0956797612441951 22961773 PMC3660998

[B21] Parkinson B. (2005). Do facial movements express emotions or communicate motives? Pers. Soc. Psychol. Rev. 9, 278–311 10.1207/s15327957pspr0904_1 16223353

[B22] Royet J. P. Koenig O. Gregoire M. C. Cinotti L. Lavenne F. Le Bars D. (1999). Functional anatomy of perceptual and semantic processing for odors. J. Cogn. Neurosci. 11, 94–109 10.1162/089892999563166 9950717

[B23] Soudry Y. Lemogne C. Malinvaud D. Consoli S.-M. Bonfils P. (2011). Olfactory system and emotion: common substrates. Eur. Ann. Otorhinolaryngol. Head Neck Dis. 128, 18–23 10.1016/j.anorl.2010.09.007 21227767

[B24] Steiner J. (1973). The gustofacial response: observation on normal and anencephalic newborn infants. Symp. Oral Sens. Percept. 4, 254–278 4612820

[B25] Terzis V. Moridis C. N. Economides A. A. (2012). The effect of emotional feedback on behavioral intention to use computer based assessment. Comput. Educ. 59, 710–721 10.1016/j.compedu.2012.03.00319019818

[B26] Wansink B. (2004). Environmental factors that increase the food intake and consumption volume of unknowing consumers. Annu. Rev. Nutr. 24, 455–479 10.1146/annurev.nutr.24.012003.132140 15189128

[B27] Zeinstra G. G. Koelen M. Colindres D. Kok F. J. de Graaf C. (2009). Facial expressions in school-aged children are a good indicator of “dislikes”, but not of “likes.” Food Qual. Prefer. 20, 620–624 10.1016/j.foodqual.2009.07.002

